# Asthma and Fixed Airways Obstruction: Real Life Aspects

**DOI:** 10.3390/arm91010007

**Published:** 2023-02-04

**Authors:** Enrico Buonamico, Andrea Portacci, Silvano Dragonieri, Vitaliano Nicola Quaranta, Fabrizio Diaferia, Elena Capozza, Luigi Macchia, Giovanna Elisiana Carpagnano

**Affiliations:** 1 Respiratory Diseases, Department “DiBraiN”, University of Bari “Aldo Moro”, 70121 Bari, Italy; 2 Allergology Unit, Department “DETO”, University of Bari “Aldo Moro”, 70121 Bari, Italy

**Keywords:** asthma, severe asthma, airways obstruction, fixed obstruction

## Abstract

**Highlights:**

**What are the main findings?**

- We found new aspects of fixed airways obstruction in severe asthma probably linked to late asthma diagnosis.
- Asthma with and without fixed airways obstruction had similar outcomes.

**What is the implication of the main finding?**

- A prompt differential diagnosis between severe asthma and COPD is essential.
- The above is important to avoid further airway remodeling.

**Abstract:**

We aimed to evaluate asthmatic patients with fixed airways obstruction (FAO) and to verify the impact of follow-up in an asthma-dedicated outpatient clinic on symptoms control and spirometry compared to asthmatics without FAO. We enrolled 20 asthmatic FAO+ patients and 20 FAO− asthmatics at baseline (T0) and at a one-year follow-up visit (T1). FAO+ and FAO− groups were compared for anamnesis, FEV1, asthma control test (ACT) and their ΔT0–T1. FAO+ and FAO− groups did not differ for age, BMI, pack-years, allergy, T0 blood eosinophils, comorbidities or GINA therapy step at T0 and T1, whereas, in the FAO+ group, we found more patients with a delay >5 years between symptoms onset and correct asthma diagnosis (*p* < 0.05). ACT at T0 and ΔT0–T1, FEV1 at ΔT0–T1 and number of exacerbations at T0 and ΔT0–T1 did not differ between groups. Despite a widespread perception of FAO, per se, as a severity factor for asthma, we found similar severity profiles and amelioration after one year of treatment in the FAO+ and FAO− groups. The only factor linked to FAO development in our population was a delay in asthma diagnosis from respiratory symptoms onset, which may have led to airway remodeling. Physicians should characterize patients with FAO for avoiding misdiagnosis between asthma and other respiratory diseases and for establishing the appropriate therapy.

## 1. Introduction

Asthma is an inflammatory chronic disease of the bronchi, which is characterized by symptoms such as cough, chest tightness, dyspnea and wheezing and pathophysiological alterations including obstructive ventilatory defect and hyperinflation, which may vary in intensity and over time [1]. Indeed, it is essential to detect the presence of reversible obstructive ventilatory defect or bronchial hyperresponsiveness to confirm a diagnosis of asthma in a suspected patient [1].

However, a small group of asthmatic patients may develop fixed airway obstruction (FAO) due to airway remodeling, which is a mechanism characterized by bronchial epithelial cells apoptosis, smooth muscle cells proliferation and fibroblast-driven extracellular matrix changes leading to persistent narrowing of small and large airways [2]; the mechanisms leading to airways remodeling are yet unclear, but seem linked to chronic inflammation through the activation of the proteolytic cascade, the synergic effect on the loss of lung elastic recoil of ageing and, in an opposite way, an early loss of lung function that may lead to a lower starting point for the normal lung development [3]. Interestingly, the above has been recently considered as one of the main negative outcomes in asthma, alongside exacerbations and systemic corticosteroids side effects [1].

Given the similar clinical and functional manifestations in asthma with FAO and COPD, it is essential to obtain a better characterization of patients with non-reversible obstructive lung disease. Indeed, asthma with FAO can be considered a type of severe asthma [3,4] and many asthmatic patients with FAO might be treated with higher doses of inhaled corticosteroids (ICS) or even biologic therapy, which has not been approved for COPD.

Based on the above, the aim of our study was to deepen our knowledge about FAO in asthma by investigating a population of patients with asthma and FAO in terms of clinical, functional and biological characteristics, compared with a population of asthmatic patients without FAO.

## 2. Materials and Methods

### 2.1. Subjects

A total of 40 patients were enrolled into this study, with an unsupervised approach.

The first group was composed of 20 consecutive patients with asthma and with fixed airflow obstruction (FAO+). FAO+ was defined as patients showing values of post-bronchodilator Forced Expiratory Volume in the 1st second/Vital Capacity (FEV1/VC) < limit of the 5th percentile (LLN) and FEV1% < 90% despite adequate control therapy, stable throughout the one-year follow-up.

The second group consisted of 20 consecutive patients with asthma and without fixed airflow obstruction (FAO−).

All patients were recruited from those attending the respiratory diseases outpatient clinic of the University hospital of Policlinico, Bari, Italy, from January 2017 to February 2020.

Inclusion criteria for both groups were diagnosis of asthma strictly following GINA guidelines and pack/year value under 10, whereas exclusion criteria for both groups were refusal of written informed consent, a more probable diagnosis of COPD (or asthma/COPD overlap) and/or any other lung diseases and/or any systemic diseases with potential lung involvement, such as Eosinophilic Granulomatosis with Polyangiitis (EGPA) or autoimmune diseases and neoplasms.

All patients gave voluntary consent to participate in the study and to process personal data.

The present study was conducted according to the World Medical Association’s 2008 Declaration of Helsinki, the guidelines for Good Clinical Practice and the “Strengthening the Reporting of Observational Studies in Epidemiology” (STROBE) guidelines [5].

In addition, this study was approved by the local Institutional Review Board (approval number: 5785).

### 2.2. Study Design

We conducted an observational prospective study. Patients were evaluated during two visits, at the first visit (T0) and at a control visit (T1) after 1 year. During this time span, patients were followed-up by our outpatient clinic, and the frequency of the visits and the prescribed therapies followed GINA guidelines according to the severity of each patient.

At both T0 and T1, all patients were investigated for respiratory symptoms onset, year of asthma diagnosis, any possible diagnosis delay (late diagnosis was considered if asthma diagnosis occurred more than 5 years from symptoms onset), common asthma comorbidities (i.e., atopy, allergic oculorhinitis, chronic rhinosinusitis with or without nasal polyps (CRswNP/CRSsNP), obesity, gastro-esophageal reflux disease (GERD), obstructive sleep apnea (OSAS)), asthma controller therapy (both inhaled and systemic) and blood eosinophils, and screened for alpha-1 antitrypsin deficiency with serum dosage. At both T0 and T1, all patients underwent a lung function test and symptoms assessment with an asthma control test (ACT). Comparisons were made between FAO+ and FAO− group for disease onset prevalence, comorbidities prevalence, naïve patients’ prevalence, FEV1 variations between T0 and T1 (intended as percentage of T0 change in volume between T1 and T0) and ACT value variations between T0 and T1. We also compared frequency distribution of positive minimally clinical important difference (MCID) in FEV1 and ACT between FAO+ and FAO− groups. An FEV1 positive MCID was considered when having an increase in FEV1 between T0 and T1 of at least 15%, whereas an ACT positive MCID was considered when having an ACT increase of at least three points between T0 and T1.

### 2.3. Lung Function

All lung function tests were performed with Jaeger™ Masterscreen Body (CareFusion, Hoechberg, Germany) following the current recommendations [6]. The following parameters were calculated: forced expiratory volume in 1st second (FEV1), forced vital capacity (FVC) and forced expiratory volume divided per vital capacity (Tiffeneau index or FEV1/VC).

### 2.4. Statistical Analysis

All continuous variables were tested for normality with a Shapiro–Wilk test.

Normal variables are shown as mean ± standard deviation (SD) and non-normal variables are shown as median (interquartile range).

Comparisons between continuous variables were tested with Student’s *t*-test for parametric variables and with a Mann–Whitney test for nonparametric variables.

Comparisons between frequencies distributions of prevalence were tested with Fisher’s exact test.

*P* values were considered significant under 0.05.

The software used for statistics was SPSS (IBM Corp. Released 2019. IBM SPSS Statistics for Windows, Version 26.0. Armonk, NY, USA: IBM Corp).

## 3. Results

**Figure 1:**
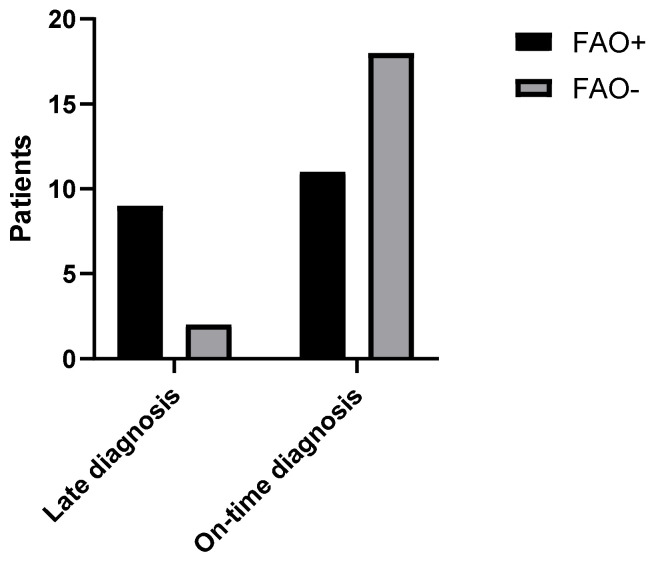
Number of patients with late-diagnosed asthma and with on-time diagnosed asthma divided per presence of FAO (FAO+ 9 pts, 45%; FAO− 2 pts, 10%; *p* < 0.05). FAO: Fixed Airways Obstruction.

Comparisons between general characteristics and comorbidity prevalence of FAO+ and FAO− group are presented in Table 1. Interestingly, patients in the FAO+ group had a greater delay in asthma diagnosis from symptoms onset (3.5 (1.25–12.25) vs. 2 (0–3.75); *p* < 0.05), and, considering the abovementioned definition of late diagnosis, nine patients (45%) in the FAO+ group received a late diagnosis of asthma, whereas this happened in only two patients (10%) of the FAO− group (*p* < 0,05, Figure 1. None of our patients had alpha-1 antitrypsin deficiency, and no participants underwent chronic systemic corticosteroid therapy.

**Figure 2:**
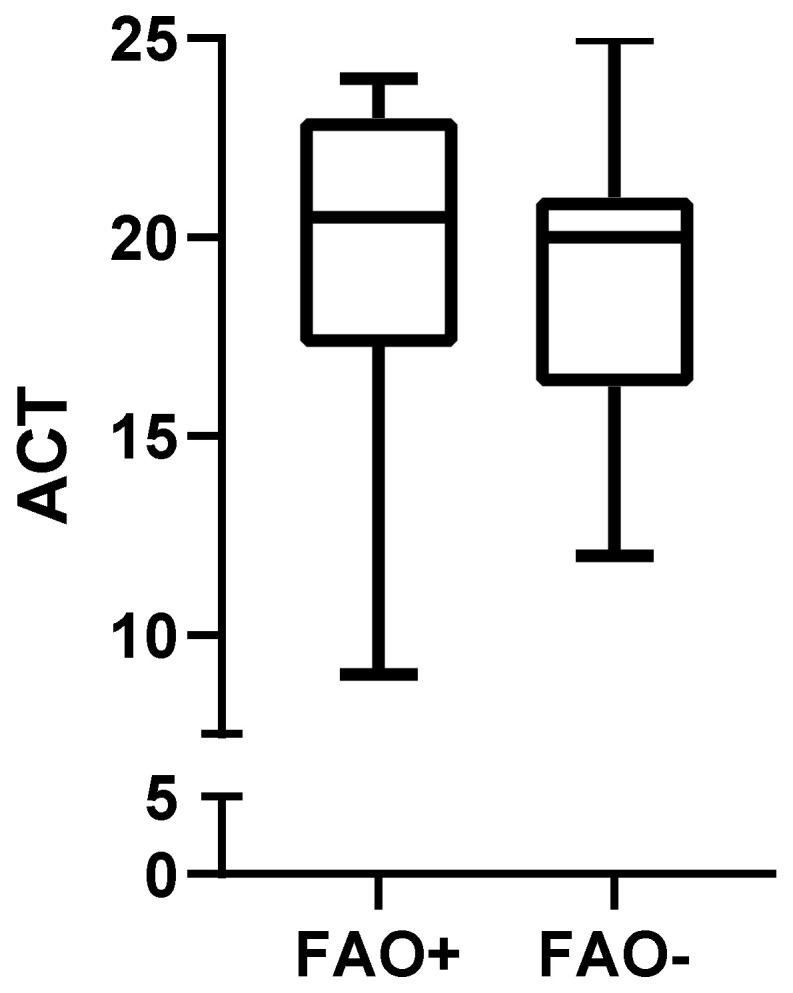
Baseline ACT in patients with FAO compared to patients without FAO (20.5 (17.25–23) vs. 20 (16.25–21); *p* = 0.24). ACT: Asthma Control Test; FAO: Fixed Airways Obstruction.

**Figure 3:**
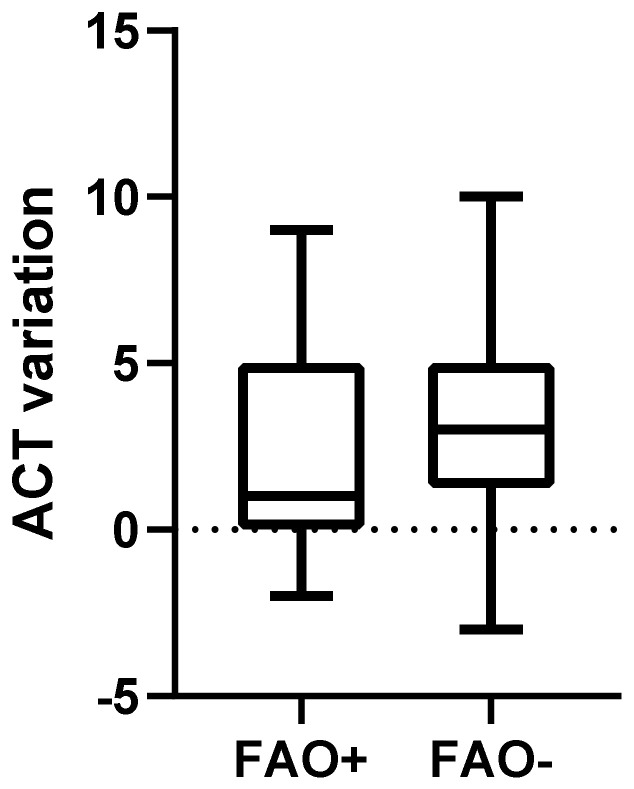
ACT variation from baseline after 1 year of follow-up in patients with FAO compared to patients without FAO (1 (0–5) vs. 3 (1.25–5); *p* = 0.13). ACT: Asthma Control Test; FAO: Fixed Airways Obstruction.

Comparison of spirometric values, symptoms evaluation and steps of therapies between FAO+ and FAO− groups at T0 and T1 are presented in Table 2. ACT at T0 did not differ significantly between FAO+ and FAO− groups (20.5 (17.25–23) vs. 20 (16.25–21); *p* = 0.24, Figure 2) and neither did ACT variation between T0 and T1 (1 (0–5) vs. 3 (1.25–5); *p* = 0.13, Figure 3). Five patients (25%) in the FAO+ group and twelve patients (60%) in the FAO− group had a positive ACT MCID and, despite not being significant, the comparison showed an important trend (*p* = 0.054).

**Figure 4:**
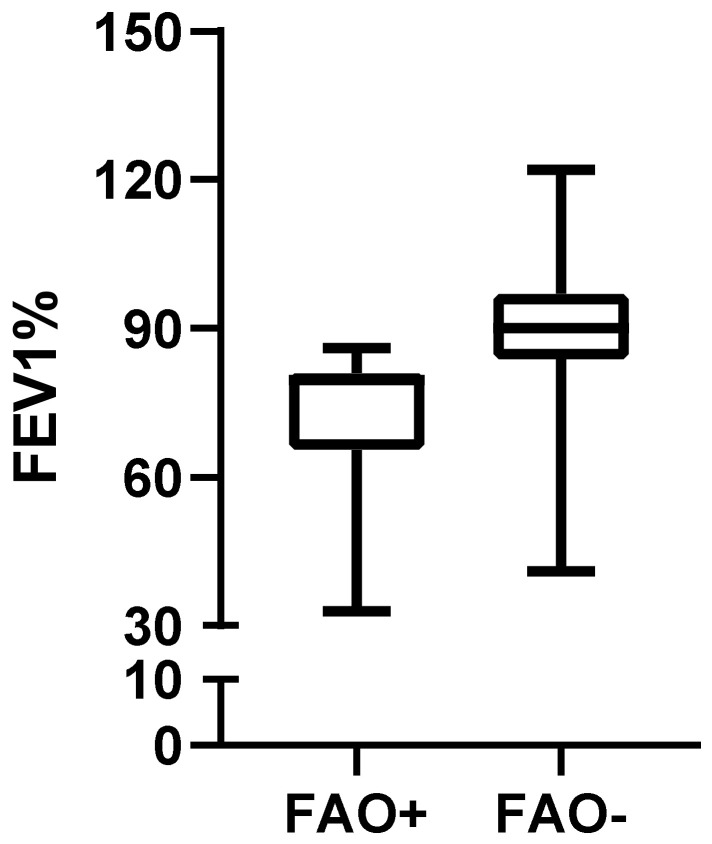
Baseline post-bronchodilator FEV1 (in percentage of theoretical values) in patients with FAO compared to patients without FAO (79.50 (65.50–81) vs. 90 (83.75–97); *p* < 0.01). FEV1: Forced Expiratory Volume 1st Second; FAO: Fixed Airways Obstruction.

**Figure 5:**
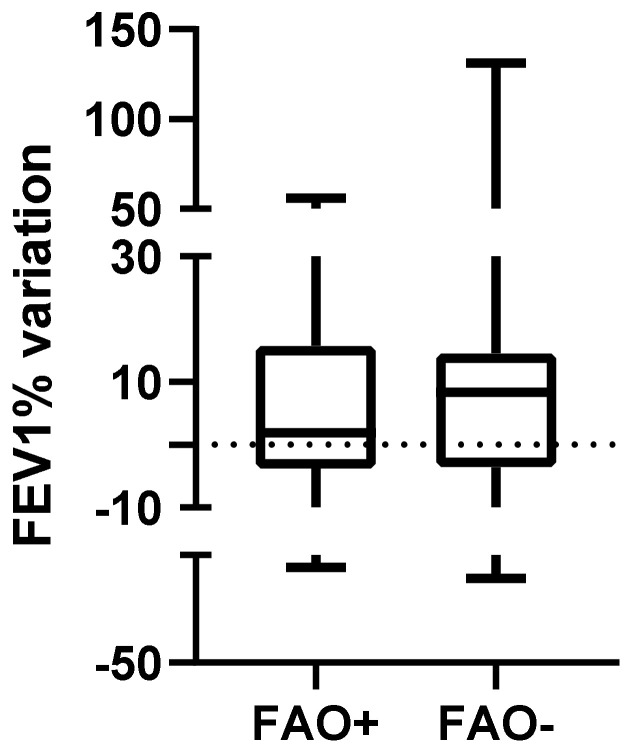
Variation of FEV1 (in percentage of theoretical values) from baseline after 1 year of follow-up in patients with FAO compared to patients without FAO (1.92 (−3.75–15.75) vs. 8.37 (−3.55–14.57); *p* = 0.66). FEV1: Forced Expiratory Volume 1st Second; FAO: Fixed Airways Obstruction.

Baseline post-bronchodilator FEV1 was, as expected, significantly lower in the FAO+ group (79.50 (65.50–81) vs. 90 (83.75–97); *p* < 0.01, Figure 4), as was T1 post-bronchodilator FEV1 (78.50 (69.5–83.75) vs. 96.5 (94–102.8); *p* < 0.01). Conversely, FEV1 variation between T0 and T1 did not differ between FAO+ and FAO− groups (1.92 (−3.75–15.75) vs. 8.37 (−3.55–14.57); *p* = 0.66, Figure 5) and positive MCID for FEV1 frequency distribution did not vary between FAO+ and FAO− groups (6 (30%) vs. 4 (20%); *p* = 0.72).

Number of last-year exacerbations at baseline did not differ between FAO+ and FAO− groups (0 (0–1) vs. 1 (0–1.75); *p* = 0.25) and the variation of last-year exacerbations number did not change significantly between groups (0 (−1–0) vs. −1 (−1–0); *p* = 0.33).

## 4. Discussion

In our study we found that, unexpectedly, the FAO+ group and the FAO− group significantly differed only for lung function, years from asthma diagnosis and ACT improvement MCID, whereas other biological, anamnestic and clinical factors were similar in the two groups.

Our groups did not significantly differ for baseline general characteristics, and, in a similar way, comparison between FAO+ and FAO− groups for blood eosinophil counts, aeroallergen sensitivity and common asthmatic comorbidities prevalence (such as type-2 inflammation-driven allergic oculorhinitis and CRswNP/CRssNP) did not show significant differences. Contrarily, in 2015 Konstantellou et al. recruited 170 asthmatic patients and identified 60 with persistent airflow limitation, and, again, through a cluster analysis, showed that the persistent airflow limitation cluster was the one with highest systemic and inhaled corticosteroid usage with highest type-2 inflammation biomarkers [7].

In addition, prevalence of GERD and OSAS, which are considered determinant comorbidities for difficult-to-control asthma, did not differ between groups. The history of tobacco exposure, expressed as P/Y, was similar in FAO+ and FAO− groups, but we chose to consider a P/Y < 10 in our inclusion criteria for general study population in order to better define asthma diagnosis, so this result is expected. Konno et al., in 2017, showed, through a cluster analysis on 127 severe asthmatic patients, that smoking is related to low FEV1 in two different clusters of patients, both T2 high and T2 low, as well as obesity [8], while our population of FAO+ patients is characterized both by very low P/Y and low BMI.

We also compared the steps of asthma therapy according to GINA guidelines between FAO+ and FAO− groups, both at baseline and after T1 visit, and we did not find any significant difference, having as well a similar median ACT between groups at baseline and a similar variation in ACT values after the one-year follow-up at our dedicated outpatient clinic. Despite this, significantly more FAO− patients presented a positive MCID for ACT from T0 to T1, showing a better feeling of symptoms amelioration in this group after the one-year follow-up. Conversely, Smith et al., in a 2019 study, demonstrated that FAO patients are characterized by higher systemic corticosteroids dependence, higher inhaled corticosteroids dosages and an overall higher severity of asthma [9].

Obviously, in the FAO+ group, baseline as well as T1 FEV1 are significantly lower than in the FAO− group, but this finding is due to the FAO+ inclusion criteria that allowed only low FEV1 patients to enter this group. On the contrary, interesting evidence is that median FEV1 variations between T0 and T1 are similar in the two groups, and, also, comparison between positive MCID in FEV1 variations show the same result, demonstrating that also patients with a more compromised lung function may benefit from a better asthma therapy setting.

Both groups were characterized by a low median number of previous-year exacerbations at baseline and a similar amelioration of this value after one year.

These findings depict a similar patient severity profile in the two groups according to symptoms control and previous-year exacerbations, and this contrasts with the diffuse belief, also found in ATS/ERS 2014 severe asthma guidelines [4], that FAO provides to asthma diagnosis severity by itself.

Interestingly, we found a trend towards a longer year from asthma diagnosis in the FAO− group and a significantly higher number of late diagnoses in the FAO+ group that could explain worse FEV1 values in the FAO+ group, considering the delay of a proper asthma control therapy setting with anti-inflammatory inhaled steroids that may have led to airway remodeling, the pathologic mechanism behind FAO.

In our opinion, the change in therapeutic strategies for COPD led by GOLD 2017 guidelines, which suggested ICS therapy only in a minor subset of COPD exacerbator patients, contributed to help proper diagnosis, especially in elderly patients with fixed airway obstruction asthma, allowing us to enrich our population of FAO+ patients with patients that, since 2017, were misdiagnosed with COPD, not characterized by evident type-2 inflammation markers or by high exacerbation number or OCS dependence, classic asthmatic stigmata.

Misdiagnosis of obstructive lung diseases has always been a hot topic, already evidenced during the past decades [10], and still is in the spotlight: Kavanagh et al., in a 2019 review, showed how asthma is underdiagnosed from 19% to 73%, and that there is a lack of data regarding the elder population, the one that suffers the most asthma-COPD misinterpretation [11].

Regarding this topic, 2014 GINA Asthma/COPD Overlap Syndrome (ACOS) guidelines [12] already tried to fix this issue, suggesting the “hybrid” diagnosis of ACOS to help clinicians to consider also asthmatic features in the proper diagnostic pathway of respiratory patients with FAO.

Our work presents some limitations, such as the scarce numerosity of our population and the short follow-up period to the lack of endotype characterization of our patients with more proper biomarkers (i.e., FeNO, blood and exhaled periostin, specific and total IgE values) apart from blood eosinophils and anamnestic determination of aeroallergen sensitization and type-2 comorbidities presence, but they are linked to the real-life setting of this study.

In conclusion, despite a widespread perception of FAO as an important, per se, characterizing severity factor for asthma, in our study, we found a similar severity profile in FAO+ and FAO− groups and a similar amelioration in symptoms control and lung function improvement (nonetheless reaching lower values in the FAO+ group) after one year follow-up at our asthma dedicated outpatient clinic. The only factor that is linked to FAO development in our population is a delay in asthma diagnosis from respiratory symptoms onset, that may have led to airway remodeling. The difference between our results and those found in the literature may be explained by the inclusion in our FAO+ group of patients that, up to 2017 GOLD COPD guidelines publications, were easily misdiagnosed as COPD. Clinicians in real-life settings, often characterized by few resources and time to dedicate to single patients, must pay particular attention to anamnesis and type-2 inflammation markers in patients presenting respiratory symptoms and not only to lung function to avoid misdiagnosis and to give asthmatic patients the right anti-inflammatory inhaled therapy that leads to important results also in this subtype of patients and may avoid further airway remodeling.

A practical approach to the problem of the misdiagnosis of asthma may be represented by the further implementation of more tests apart from spirometry in respiratory patients with FAO, for example, the evaluation of easily obtainable type-2 biomarkers such as fraction exhaled nitric oxide (FeNO) or peripheral blood eosinophil count, as proposed by Paraskevi et al., in children patients [13].

## Figures and Tables

**Table 1:** Comparisons between general characteristics and comorbidities of FAO+ and FAO− groups.

Parameter	FAO+	FAO−	*p*
Age	51 (44.25–68)	57 (32–66)	0.93
BMI	25.85 ± 3.79	26.85 ± 4.93	0.48
P/Y	0 (0–7.25)	0 (0–7.87)	0.77
Early onset asthma	5 (25%)	3 (15%)	0.69
Years from respiratory symptoms onset	12 (8–21)	12 (6.25–27.25)	0.89
Years from asthma diagnosis	5 (0.5–11.75)	10 (3–22.75)	0.06
Years of delay between symptoms onset and diagnosis	3.5 (1.25–12.25)	2 (0–3.75)	<0.05
Late diagnosis	9 (45%)	2 (10%)	<0.05
Baseline blood eosinophils	256 (±145)	285 (±134)	0.51
Aeroallergen sensitivity	16 (80%)	13 (65%)	0.48
Allergic oculorhinitis	11 (55%)	9 (45%)	0.75
CRswNP/CRssNP	8 (40%)	3 (15%)	0.15
GERD	8 (40%)	8 (40%)	1
OSAS	3 (7.5%)	3 (7.5%)	1

**Table 2:** Comparisons between spirometric values, asthma control and therapy steps between FAO+ and FAO− groups at T0 and T1.

Parameter	FAO+	FAO−	*p*
FEV1% (baseline)	79.50 (65.50–81)	90 (83.75–97)	<0.01
FEV1% (T1)	78.50 (69.5–83.75)	96.5 (94–102.8)	<0.01
FEV1% variation between T1 and T0	1.92 (−3.75–15.73)	8.37 (−3.55–14.57)	0.65
FVC% (baseline)	89.30 ± 18.12	103.7 ± 11.95	<0.01
FVC% (T1)	92.25 ± 12.48	109.2 ± 10.72	<0.01
FVC% variation between T1 and T0	0.12 (−4.91–11.44)	3.355 (0.19–8.82)	0.22
ACT (T0)	20.5 (17.25–23)	20 (16.25–21)	0.24
ACT (T1)	23 (20.25–24)	22.5 (21–23.75)	0.6
ACT variation between T1 and T0	1 (0–5)	3 (1.25–5)	0.13
GINA step therapy at baseline			0.81
No therapy	5 (25%)	5 (25%)	
Step 1	1 (5%)	1 (5%)	
Step 2	0	1 (5%)	
Step 3	10 (50%)	11 (55%)	
Step 4	3 (15%)	2 (10%)	
Step 5	1 (5%)	0	
GINA step therapy at T1			0.54
Step 3	14 (70%)	16 (80%)	
Step 4	5 (25%)	4 (20%)	
Step 5	1 (5%)	0	
Chronic oral steroid assumption	0	0	

## Data Availability

Available at reasonable request.
